# Enhanced Control of Nonlinear Systems Under Control Input Constraints and Faults: A Neural Network-Based Integral Fuzzy Sliding Mode Approach

**DOI:** 10.3390/e26121078

**Published:** 2024-12-10

**Authors:** Guangyi Yang, Stelios Bekiros, Qijia Yao, Jun Mou, Ayman A. Aly, Osama R. Sayed

**Affiliations:** 1 Information Center, Hunan Institute of Metrology and Test, Changsha 410014, China; guangyiyang_himt@163.com; 2 Department of Management, University of Turin (UniTo), 10134 Turin, Italy; 3 School of Automation and Electrical Engineering, University of Science and Technology Beijing, Beijing 100083, China; qijia_yao@ustb.edu.cn; 4 School of Information Science and Engineering, Dalian Polytechnic University, Dalian 116034, China; moujun@csu.edu.cn; 5 Department of Mechanical Engineering, College of Engineering, Taif University, Taif 21944, Saudi Arabia; aymanaly@tu.edu.sa; 6 Department of Mathematics, Faculty of Science, Assiut University, Assiut 71516, Egypt; o_sayed@aun.edu.eg

**Keywords:** neural network estimator, fuzzy logic, finite-time stability, integral sliding surface, faults control, control input constraints

## Abstract

Many existing control techniques proposed in the literature tend to overlook faults and physical limitations in the systems, which significantly restricts their applicability to practical, real-world systems. Consequently, there is an urgent necessity to advance the control and synchronization of such systems in real-world scenarios, specifically when faced with the challenges posed by faults and physical limitations in their control actuators. Motivated by this, our study unveils an innovative control approach that combines a neural network-based sliding mode algorithm with fuzzy logic systems to handle nonlinear systems. This proposed controller is further enhanced with an intelligent observer that takes into account potential faults and limitations in the control actuator, and it integrates a fuzzy logic engine to regulate its operations, thus reducing system chatter and increasing its adaptability. This strategy enables the system to maintain regulation in the face of control input constraints and faults and ensures that the closed-loop system will achieve convergence within a finite-time frame. The detailed explanation of the control design confirms its finite-time stability. The robust performance of the proposed controller applied to autonomous and non-autonomous systems grappling with control input limitations and faults demonstrates its effectiveness.

## 1. Introduction

Control of nonlinear systems has been a pivotal subject in control theory, and its importance has been extensively addressed in the literature [1,2]. Nonlinear systems are inherent in various fields, including physics, engineering, economics, biology, and many others, encompassing a vast range of real-world applications. As such, nonlinear systems have been at the center of numerous theoretical and practical investigations leading to several groundbreaking results and methodologies [3,4]. Theoretical efforts have spanned from Lyapunov’s second method to backstepping, while practical methodologies mostly have ranged from gain scheduling to sliding mode control. These solutions, in many instances, have successfully addressed the complexities of nonlinearities, yet it is a well-accepted notion that the problem is far from being completely solved [5,6]. The very nature of nonlinear systems, characterized by high dimensionality, time-varying parameters, hysteresis, and other inherent complexities, makes them an active field of research.

Despite the promising progress made in the field, several challenging issues persist. The presence of faults and limitations in the control inputs poses significant complications that require dedicated attention [7,8]. Real-world systems often face limitations such as physical constraints, noise interference, actuator faults, sensor failures, and many others that impair effective control of the systems. These difficulties are frequently neglected in the development of control techniques, where an ideal, fault-free environment is often assumed [9,10,11]. It is imperative to recognize that certain assumptions may not adequately address the complexities of real-world practicalities, wherein components are subject to wear and tear, external disturbances, and environmental variances. Consequently, it is of paramount importance for research endeavors to emphasize the development of controllers that encapsulate these considerations, thereby ensuring heightened robustness and reliability in practical implementations.

A second, yet equally challenging issue, is the phenomenon of chattering and vibration in control systems [12,13,14]. Chattering, in particular, is a well-documented problem that manifests as undesirable high-frequency oscillations in the control action. Even renowned control strategies such as sliding mode control are susceptible to this issue [15,16]. The implications of chattering extend beyond control performance, leading to damaging effects on system components and reducing the operational lifespan. The issue becomes even more pronounced in high-frequency control applications, demanding concerted research efforts toward the development of control strategies that effectively mitigate chattering while ensuring optimal system performance.

Neural networks have introduced innovative approaches across various technologies [17,18,19,20]. Specifically, neural networks have shown promise in various control applications due to their ability to learn complex dynamics from data [21,22,23]. These networks have demonstrated remarkable capabilities in adaptively controlling nonlinear systems, with their internal states being able to process and remember past inputs, making them suitable for sequential data processing [24,25]. Their ability to handle nonlinearity and learn from data presents a new wave of solutions that can revolutionize control system design and implementation.

In response to the aforementioned issues, this paper proposes a novel control strategy—a Neural Network-based Fuzzy Integral Sliding Mode Control. This approach integrates the powerful learning capability of neural networks with the interpretability and adaptability of fuzzy systems to offer a robust solution against faults and failures in control inputs and prevent chattering in system response. The fuzzy logic system in this approach adapts the control strategy in real time, thereby ensuring a smooth system response. In addition, this proposed control method guarantees finite-time convergence, an attribute of paramount importance in practical applications where timely system responses are imperative. In our proposed strategy, we continually update both the weights and biases of the feedforward neural networks through an adaptive learning mechanism, allowing the network to learn and adjust to the system dynamics effectively. Concurrently, we employ a fuzzy logic system with rules designed to prevent chattering and ensure a smooth system response. In summary, the proposed control method offers two main key contributions: (1) Enhanced Fault Tolerance and Robustness: The integration of neural networks allows the control system to continuously learn and adapt to the system dynamics, effectively compensating for faults and uncertainties in real time. (2) Smooth and Stable System Response: The fuzzy logic component dynamically adjusts the control strategy to mitigate high-frequency oscillations and chattering, ensuring a smooth and stable system response.

The article is organized as follows: Section 2 introduces the synchronization challenge faced during faults and control input constraints and details the design of the deep neural network estimator. Section 3 discusses the controller design methodology, offers stability proof, and introduces our method for integrating a fuzzy engine. In Section 4, we evaluate the controller’s efficacy on chaotic nonlinear autonomous and non-autonomous systems. The article wraps up in Section 5 with a recap of key findings and potential avenues for future research.

## 2. Modeling Control Input Challenges and Neural Network Estimator

Consider the state space equation of the slave system given in the following general form:

(1) $${\dot{x}}_{i}\left(t\right)=\frac{1}{g_{i}\left(x,t\right)}\left(f_{i}\left(x,t\right)+u_{i}\right)\;\;i=1,2,\ldots ,n$$

The state of this slave system is represented by $x_{i}$, where *i* spans from 1 to n. The system’s uncertain nonlinear function is portrayed by $f_{i}\left(x,t\right)$ and $g_{i}\left(x,t\right)$, while $u_{i}$ represents the control input. In addition, the master system is characterized as follows:

(2) $$D_{t}^{q}y_{i}=h_{i}\left(y,t\right)\;\;(i=1,2,3,4)$$

within this framework, the states of the master system are represented by y, and the nonlinear functions characterizing the system are denoted by $h_{i}\left(y,t\right)$.

Note that the master system refers to the desired reference model that defines the target dynamics and ideal behavior of the system. It serves as a baseline or reference trajectory for the controlled system. Conversely, the slave system represents the actual system that may be subject to faults, uncertainties, or perturbations. The goal of the control strategy is to ensure that the slave system follows and synchronizes with the master system, thereby achieving the desired performance despite any deviations or disturbances.

### 2.1. Faults and Control Input Constraints

In practical engineering systems, there exists an inherent vulnerability to faults or failures that, if unchecked, can compromise system functionality or culminate in complete system breakdown. Consequently, the design and implementation of fault-tolerant control strategies that can efficaciously identify and mitigate the repercussions of these unforeseen disturbances become imperative. Drawing inspiration from the concept of actuator faults, coupled with the acknowledgment of physical limitations on the control input in reference [26,27], we propose the subsequent model to account for both faults and the inherent restrictions in the control input:

(3) $$u_{i}=\left\{\begin{array}{c} u_{i\_max}\;\;\;\;\;\;\;\;\;\;\;\;\;\;\;\;\;\;\;\;\;\;\;\;\;\;\;\;\;\;\;\;\;\;\;\;\;\;\;\;\;\;\;\;\;\;\;\;\;\;\;\;\;\;\;\;\;\;\;\;\;\;\;\;\;\;\;\;\;\mathrm{if}\;\;\;u_{i\_a}>u_{i\_max}\\ u_{i\_a}+a_{i}\left(t\right)\left(\left(c_{i}-1\right)u_{i\_a}+{\bar{u}}_{i}\right)\;\;\;\;\;\;\;\;\;\;\;\;\;\;\;\;\;\;\;\;\;\;\mathrm{if}\;\;u_{i\_min}<u_{i\_a}<u_{i\_max}\\ u_{i\_min}\;\;\;\;\;\;\;\;\;\;\;\;\;\;\;\;\;\;\;\;\;\;\;\;\;\;\;\;\;\;\;\;\;\;\;\;\;\;\;\;\;\;\;\;\;\;\;\;\;\;\;\;\;\;\;\;\;\;\;\;\;\;\;\;\;\;\;\;\;\;\;\mathrm{if}\;\;\;\;\;\;u_{i\_a}<u_{i\_min} \end{array}\right.$$

In this formulation, $u_{i\_min}$ and $u_{i\_max}$ are the minimum and maximum constraints the actuator enforces on the system. Additionally, the symbols ${\bar{u}}_{i}$, $u_{i\_a}$, and $u_{i}$ are employed to signify the variable constant fault input, intended control input, and real control input correspondingly. The parameter that lies within the range of 0 ≤ $c_{i}$ ≤ 1 is used to signify the efficiency of the actuator control. The time-variant function $a_{i}\left(t\right)$ represents the time-sensitive nature of a fault that affects the actuator. This can be formulated as follows:

(4) $$a_{i}(t)=\left\{\begin{array}{c} 0,\;\;\;\;\;\;\;\;\;\;\;\;\;\;\;\;\;\;\;\;\;\;\;\;\;\;\;\;t<t_{0}\\ 1-e_{i}^{-b_{i}\left(t-t_{0}\right)}\;\;\;\;\;\;\;t\geq t_{0} \end{array}\right.$$

In this context, the parameter $b_{i}>0$ represents the unknown rate of fault progression, while marks the instance of the fault occurrence. The ensuing equation depicts the state space equation of the system, which embodies the potential for the occurrence of actuator faults and/or failures:

(5) $$\begin{array}{c} {\dot{x}}_{i}=f_{i}\left(x,t\right)+g_{i}\left(x,t\right)u_{i}+\theta_{i}\left(t\right),\;\;\;\;\;\;\;\;\;\;i=1,2,...,n-1\\ \theta_{i}(t)=a_{i}(t)\left(\left(c_{i}-1\right)u_{i\_a}+{\bar{u}}_{i}\right) \end{array}$$

**Remark** **1.** *The initial system formulation in Equation (1) does not incorporate external disturbances or uncertainties, as the focus is on developing a robust control strategy for handling faults and constraints in control inputs. However, the complexity in the proposed approach arises from managing the internal uncertainties and unknown parameters introduced by control actuator faults, as captured in Equations (3)–(5). These internal uncertainties significantly impact system stability and performance, making the control design more challenging and necessitating the use of advanced control methods*.

### 2.2. Neural Network Estimator

Neural networks have dramatically revolutionized the field of control systems. They have introduced a new level of dynamism and adaptability, enabling systems to learn and adjust to changing circumstances, making them more robust and efficient. Their ability to learn complex, nonlinear system behaviors and predict future states based on historical data is particularly significant [28,29]. This has resulted in more precise and adaptive control strategies that can handle a variety of real-world complexities, thus boosting performance and reliability. Furthermore, neural networks have also been instrumental in handling uncertainties and disturbances that are commonly encountered in control systems, thereby greatly enhancing system stability. By enabling fault detection and isolation, as well as the optimization of control strategies, neural networks have indeed brought about a transformative change in the landscape of control systems [30,31,32].

In the current study, we harness the capabilities of neural networks for fault detection in complex nonlinear systems. The intrinsic nonlinear nature of neural networks makes them aptly suited for representing and analyzing behaviors typical of real-world systems. Their superior pattern recognition capabilities mean that they excel at pinpointing intricate deviations in data, often indicative of faults. This is especially valuable as faults often manifest as subtle anomalies, easily overlooked by traditional methods. Moreover, the adaptability of neural networks ensures that as they are fed continuous data, their models evolve, accounting for the system’s dynamic nature and potential emerging fault patterns.

As systems burgeon in complexity, traditional fault detection methods can become overwhelmed by the data deluge [33,34]. Neural networks, however, remain undaunted, effectively scaling with increasing data dimensions. Paired with advancements in computational hardware, they can offer real-time data processing, ensuring that faults are detected promptly—a crucial feature to preempt potential system catastrophes. Given these strengths, our research aims to leverage neural networks in setting a new gold standard for fault detection methodologies in nonlinear systems.

Through the definition of $\zeta_{i}$ as an input vector, alongside ${\hat{\theta }}_{i}$ functioning as a predictive model for disturbances, which accounts for a comprehensive range of uncertainties and fluctuations, the output derived from the feedforward neural networks can be represented in the subsequent form:

(6) $${\hat{\theta }}_{i}={\hat{W_{i}}}^{T}\zeta_{i}+b_{i}+\varepsilon =\theta_{i}+\varepsilon$$

In this context, the weight vector, denoted as $\hat{W_{i}}$, arranged in a column-like structure as ${\left[\varpi_{1},\varpi_{2},\ldots ,\varpi_{n}\right]}^{T}$, play an important role and should be carefully learned and updated. In addition, the bias term is denoted as $b_{i}$, and the estimation error is symbolized by *ε*.

## 3. Controller Design and Its Finite-Time Stability

In this section, we delineate our proposed approach. Section 3.1 delves into the intricacies of the control law. We elaborate on its finite-time stability and convergence properties, shedding light on the mathematical underpinnings and their implications in real-world scenarios. Proceeding to Section 3.2, we pivot our focus towards capitalizing on the power of fuzzy logic. Here, we infuse the control law with the finesse of fuzzy logic, integrating fuzzy terms, membership functions, and a comprehensive fuzzy rule base. This amalgamation is aimed at bolstering the control system’s adaptability.

### 3.1. Fault-Tolerant Integral Sliding Mode Control

Now, we leverage the integral terminal sliding mode technique and feedforward neural network methodologies to achieve the control objective $\underset{t\to \infty }{lim}x_{i}(t)=y_{i}(t)$ for nonlinear systems under the influence of control input constraints and faults. The discrepancy between the states of the slave system and those of the master system, defined as $e_{i}(t)=x_{i}(t)-y_{i}(t)$, is referred to as the synchronization error.

Incorporating an integral term into the sliding surface allows the system states to begin motion directly on the sliding surface from any initial conditions, thus eliminating the reaching phase typical of conventional SMC [35]. In the development of the integral terminal sliding mode tracking controller, we define the following sliding surface:

(7) $$s_{i}(t)=e_{i}(t)+\beta \int_{0}^{t}e_{i}^{\omega_{1}/\omega_{2}}(\tau )d\tau \;\mathrm{and}\;s_{i}\left(0\right)=0$$

here, $\omega_{1}$ and $\omega_{2}$ are odd integers with $\omega_{1}>\omega_{2}>0$. Additionally, β is a positive design parameter. When situated on the surface $s_{i}(t)=0$, Equation (7) is equivalent to the following:

(8) $$e_{i}\left(t\right)=-\beta \int_{0}^{t}e_{i}^{\omega_{1}/\omega_{2}}\left(\tau \right)d\tau$$

The first-order derivative of Equation (8) is provided as follows:

(9) $${\dot{e}}_{i}\left(t\right)=-\beta e_{i}^{\omega_{1}/\omega_{2}}$$

Upon integrating both sides of the equation, we obtain the following result:

(10) $$\beta \int_{T_{i}}^{T_{f}}d\tau =-\int_{e_{i}(T_{i})}^{e_{i}(T_{f})}\frac{1}{e_{i}^{\omega_{1}/\omega_{2}}}de$$

By solving Equation (10), we can determine the time of convergence for $e_{i}(t)$ as follows:

(11) $$T_{f}=T_{i}+\frac{e_{i}{\left(T_{i}\right)}^{1-\omega_{1}/\omega_{2}}}{\beta^{1-\omega_{1}/\omega_{2}}(1-\frac{q}{p})}=\frac{e_{i}{\left(T_{i}\right)}^{1-\omega_{1}/\omega_{2}}}{\beta^{1-\omega_{1}/\omega_{2}}(1-\frac{q}{p})}$$

Upon differentiating $s_{i}(t)$ with respect to time, given the dynamics expressed in Equation (1), we obtain the following:

(12) $${\dot{s}}_{i}\left(t\right)={\dot{e}}_{i}+\beta e_{i}^{\omega_{1}/\omega_{2}}\left(t\right)=f_{i}\left(x\right)+g_{i}\left(x\right)u_{i}+\theta_{i}(x)-{\dot{y}}_{i}+\beta {e_{i}}^{\omega_{1}/\omega_{2}}\left(t\right)$$

The feedforward neural network-based robust integral terminal sliding mode technique is proposed as follows:

(13) $$u_{i}=\frac{1}{g_{i}(x,t)}\left({\dot{y}}_{i}-f_{i}\left(x,t\right)-\left(\alpha +\gamma \left|e_{i}^{\frac{\omega_{1}}{\omega_{2}}}\left(t\right)\right|\right)sign\left(s_{i}\right)-{\hat{\theta }}_{i}\right)$$

Here, α, γ, and α represent positive design parameters, subject to the conditions γ>β and α being positive. Note that the time derivative ${\dot{y}}_{i}$ can either be provided directly or computed using the function h, as described in Equation (2). For control purposes, knowing ${\dot{y}}_{i}$ is sufficient, eliminating the need for explicit knowledge of h. Additionally, the discrepancy between the estimated weights (${\hat{W}}_{i}$) and the actual/ground truth weights ($W_{i}^{*}$) is characterized as the weight estimation error, which can be represented as follows:

(14) $${\tilde{W}}_{i}=W_{i}^{*}-{\hat{W}}_{i}$$

The approach proposed for adjusting the weights of the neural network can be articulated as an adaptation law, delineated by the following equation:

(15) $${\dot{\hat{W}}}_{i}=-\xi_{i}s_{i}\zeta_{i}$$

In this context, $\xi_{i}$ stands for a design parameter that holds a positive value.

**Theorem** **1.** *By implementing the control law proposed in Equation (13), all signals of the closed-loop system (1) subject to control fault and constraint (3) converge to the desired value within a finite timeframe.*

**Proof.**  We select the following expression as the candidate for the Lyapunov function:

(16) $$V_{0}=\sum_{i=1}^{n}\frac{1}{2}s_{i}^{2}+\sum_{i=1}^{n}\frac{1}{2\xi_{i}}{\tilde{W}}_{i}^{2}$$

Differentiating the Lyapunov function candidate with respect to time yields the following:

(17) $$\begin{array}{cc} {\dot{V}}_{2}=\sum_{i=1}^{n}s_{i}{\dot{s}}_{i} & +\frac{1}{\xi_{i}}{\dot{\tilde{W}}}_{i}{\tilde{W}}_{i}\\ & =\sum_{i=1}^{n}s_{i}({\dot{e}}_{i}+\beta e_{i}^{\frac{\omega_{1}}{\omega_{2}}}\left(t\right)\\ & =f_{i}\left(x\right)+g_{i}\left(x\right)u_{i}\\ & +\theta_{i}(x)-{\dot{y}}_{i}+\alpha {e_{i}}^{\frac{\omega_{1}}{\omega_{2}}}\left(t\right))+\frac{1}{\xi_{i}}{\dot{\tilde{W}}}_{i}{\tilde{W}}_{i} \end{array}$$

We now implement the proposed control signal (13).

(18) $${\dot{V}}_{2}=\sum_{i=1}^{n}s_{i}(f\left(x\right)+\left({\dot{x}}_{d}-f\left(x\right)-\left(\alpha +\gamma \left|e_{i}^{\frac{\omega_{1}}{\omega_{2}}}\left(t\right)\right|\right)sign\left(s_{i}\right)-{\tilde{\theta }}_{i}\right)-{\dot{y}}_{i}+\beta {e_{i}}^{\frac{\omega_{1}}{\omega_{2}}}\left(t\right))+\frac{1}{\xi_{i}}{\dot{\tilde{W}}}_{i}{\tilde{W}}_{i}$$

As per Equations (6) and (14), ${\tilde{\theta }}_{i}={\tilde{W}}_{i}\zeta_{i}+\varepsilon$. Consequently, we have the following:

(19) $$\sum_{i=1}^{n}s_{i}\left(-\left(\alpha +\gamma \left|e_{i}^{\frac{\omega_{1}}{\omega_{2}}}\left(t\right)\right|\right)sign\left(s_{i}\right)-{\tilde{W}}_{i}\zeta_{i}-\varepsilon +\beta e_{i}^{\frac{\omega_{1}}{\omega_{2}}}\left(t\right)\right)+s_{i}\zeta_{i}{\tilde{W}}_{i}\;=\sum_{i=1}^{n}-s_{i}\left(\alpha +\gamma \left|e_{i}^{\frac{\omega_{1}}{\omega_{2}}}\left(t\right)\right|\right)sign\left(s_{i}\right)-\varepsilon s_{i}+s_{i}\beta e_{i}^{\frac{\omega_{1}}{\omega_{2}}}\left(t\right)$$

Given the design parameters β, γ, and α are positive, and γ>β, we derive the following:

(20) $$s_{i}{\dot{s}}_{i}\leq -\left(\alpha \right)\left|s_{i}\right|-\varepsilon s_{i}$$

Given that $\alpha >\left|\varepsilon \right|$, we arrive at the following conclusion:

(21) $$s_{i}{\dot{s}}_{i}\leq 0$$

Given the conditions stipulated in Equation (21), the formulated control scheme adheres to the Lyapunov condition. As a result, the tracking error e is confirmed to asymptotically approach zero. □

### 3.2. Literature Review of Stability Concepts in Control Systems

Note that the results in Equation (11) demonstrate the finite-time convergence of the error dynamics, assuming $s_{i}\left(0\right)=0$, indicating the time required for the error $e_{i}$ to reach zero. However, the sliding surface $s_{i}\left(t\right)$ itself converges exponentially, as proven in Equation (21). While exponential convergence is sufficient in many scenarios, applying finite-time convergence techniques to the sliding surface could further improve the overall system performance and robustness, making it an interesting direction for future research. In what follows, we briefly review stability concepts in control systems.

The stability of control systems has been a central topic in control theory, with various stability concepts developed to address specific system requirements. Traditional asymptotic stability ensures that the system states gradually approach equilibrium over time, but it does not offer any guarantees regarding the speed or time required for convergence. This limitation has led to the exploration of advanced stability notions, such as exponential stability, finite-time stability, fixed-time stability, and prescribed-time stability, which provide more control over the convergence characteristics of the system.

Exponential stability guarantees that the states of a system decay towards equilibrium at an exponential rate, characterized by the decay rate of a Lyapunov function. This concept provides a faster convergence rate compared to asymptotic stability, but it does not provide a precise bound on the time required to reach a specific neighborhood of the desired state [36]. To address this limitation, finite-time stability was introduced. Finite-time stability ensures that the system states converge to the equilibrium point within a finite time, which is a function of the initial conditions [37]. This property is particularly useful in scenarios requiring rapid stabilization. However, the sensitivity of finite-time stability to initial conditions and disturbances can limit its applicability. To overcome the limitations of finite-time stability, the concept of fixed-time stability was developed [38]. Fixed-time stability guarantees that the convergence time is bounded and independent of the initial conditions, providing a more robust solution in the presence of uncertainties. This property makes fixed-time stability a suitable choice for applications requiring uniform convergence times across all initial states, such as robotic systems and high-precision tracking controllers.

A more recent advancement is the notion of prescribed-time stability, where the convergence time can be predefined by the designer, offering greater flexibility and control over the system’s transient response [39]. This approach allows control designers to achieve a desired stabilization time, regardless of the system’s initial conditions, making it highly effective for applications with stringent time constraints. By specifying a convergence time beforehand, prescribed-time stability provides a powerful tool for designing controllers in applications that require predictable and consistent convergence behavior.

Incorporating these advanced stability concepts in control design has shown significant improvements in system performance, especially in fault-tolerant control and complex nonlinear systems [40,41,42]. Their application in scenarios with constraints and uncertainties has made them a valuable addition to modern control methodologies. Leveraging these properties enables control systems to achieve precise, robust, and rapid stabilization, meeting the demands of real-world engineering applications. In the present study, we employ finite-time convergence to ensure that the system trajectories reach the sliding surface within a finite duration. Once on the sliding surface, we take advantage of exponential convergence to drive the states to the desired trajectory. This combination of finite-time and exponential convergence ensures both rapid stabilization of the sliding surface and smooth tracking behavior along the surface, resulting in enhanced system performance and robustness.

### 3.3. Fuzzy Fault-Tolerant Integral Sliding Mode Control

One of the significant challenges when designing sliding mode control pertains to the chattering phenomenon, as outlined in many research studies [43,44,45]. To reduce chattering, we replace the sign function with the output of a fuzzy logic system, $F_{s}$, which smoothly transitions between control inputs. This system is defined by a set of membership functions and fuzzy rules that approximate the behavior of $sign(s_{i})$. The proposed fuzzy mechanism maps the input variables $s\;and\;\dot{s}$ to output variable $F_{s}$. Here, $F_{s}$ is utilized in lieu of the discontinuous function sign(.). Ultimately, the proposed fuzzy fault-tolerant integral sliding mode control is presented as follows:

(22) $$u_{i}=g_{i}^{-1}(x)\left({\dot{y}}_{i}-f_{i}\left(x\right)-\left(\alpha +\gamma \left|e_{i}^{\frac{\omega_{1}}{\omega_{2}}}\left(t\right)\right|I\right)F_{s}-{\hat{\theta }}_{i}\right)$$

Herein, $F_{s}$ and is procured from the fuzzy logic engine, and the remaining parameters are designed to meet the stability condition. The sign of $F_{s}$ is aligned with sign ($s_{i}$) in accordance with the stability conditions of the designed fault-tolerant integral sliding mode control, dictating whether $F_{s}$ is negative or positive.

The fuzzy logic system uses Gaussian membership functions for the input variables $s_{i}$:

(23) $$\mu_{\mathrm{NB}}\left(s_{i}\right)=\exp\left(-\frac{{\left(s_{i}+1\right)}^{2}}{2\sigma^{2}}\right)\;\mu_{N}\left(s_{i}\right)=\exp\left(-\frac{{\left(s_{i}+0.5\right)}^{2}}{2\sigma^{2}}\right)\;\mu_{Z}\left(s_{i}\right)=\exp\left(-\frac{{s_{i}}^{2}}{2\sigma^{2}}\right)\;\mu_{P}\left(s_{i}\right)=\exp\left(-\frac{{\left(s_{i}-0.5\right)}^{2}}{2\sigma^{2}}\right)\;\mu_{\mathrm{PB}}\left(s_{i}\right)=\exp\left(-\frac{{\left(s_{i}-1\right)}^{2}}{2\sigma^{2}}\right)$$

**Figure 1:**
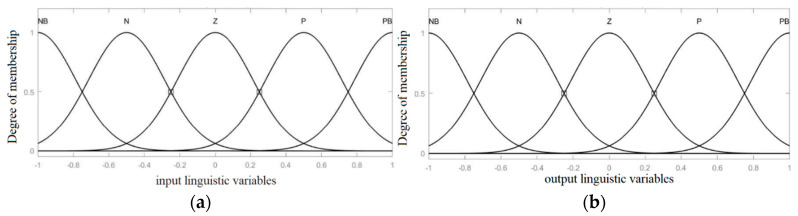
Membership functions of (**a**) input variables $s\;and\;\dot{s},$ (**b**) $output\;linguistic\;variables\;F_{s}$.

Here, σ controls the width of the Gaussian functions. The same is used for input variable ${\dot{s}}_{i}$.The fuzzy system partitions $F_{s},\;s_{i},$ and ${\dot{s}}_{i}$ into five distinct sets: Positive Big (PB), Positive (P), Zero (Z), Negative (N), and Negative Big (NB). These sets are normalized to fit within the range of −1 to 1. In this study, the fuzzy system is designed using Mamdani’s minimum operator, with the Min operator used for conjunction and the Max operator applied for rule aggregation. Gaussian membership functions, used for the fuzzy controller, are illustrated in Figure 1. The rule base used for the fuzzy inference engine is shown in Table 1. The fuzzy output $F_{s}$ can be expressed as follows:

(24) $$F_{s}=\frac{\sum_{k=1}^{N}\mu_{k}\left(s_{i},\;{\dot{s}}_{i}\right)\cdot f_{k}}{\sum_{k=1}^{N}\mu_{k}\left(s_{i},\;{\dot{s}}_{i}\right)}$$

where $\mu_{k}\left(s_{i},\;{\dot{s}}_{i}\right)$ are the membership functions, which map the sliding variable $\left(s_{i}\right)$ and its derivative $\left({\dot{s}}_{i}\right)$ to fuzzy degrees of truth. $f_{k}$ are the output values corresponding to the fuzzy rules (e.g., −1, 0, or 1). N is the number of fuzzy rules.

Instead of switching abruptly between ±1, as in the sign function, the fuzzy system produces intermediate values between −1 and 1 based on the value of $s_{i}$. This smooth transition reduces high-frequency oscillations. Mathematically, the change in $F_{s}$ with respect to $s_{i}$ is smoother than that of the sign function:

(25a) $$\frac{d}{ds_{i}}sign(s_{i})\to \infty (discontinuous\;switching)$$

(25b) $$\frac{d}{ds_{i}}F_{s}\to finite\;value(smooth\;transition)$$

By incorporating both $s_{i},$ and ${\dot{s}}_{i}$, the fuzzy system adapts the control input $u_{i}$ dynamically, resulting in smoother transitions compared to conventional methods that rely solely on the $sign(s_{i})$ function. This smoothing effect reduces high-frequency oscillations and chattering, leading to a more stable control response.

The fuzzy rule base in Table 1 provides the relationship between $s_{i},$ and ${\dot{s}}_{i}$ to determine $F_{s}$. For example, when $s_{i}$ is Positive Big (PB) and is Positive (P), $F_{s}$ is set to Positive Big (PB). This rule base ensures that the fuzzy system can handle various scenarios based on the position and rate of change of the error dynamics.

By employing these labels and functions, we establish a coherent and organized system for our fuzzy logic engine, which greatly assists in the design and optimization process. The specific fuzzy rules that are applied throughout this study are comprehensively documented in Table 1 for ease of reference and understanding.

**Figure 2:**
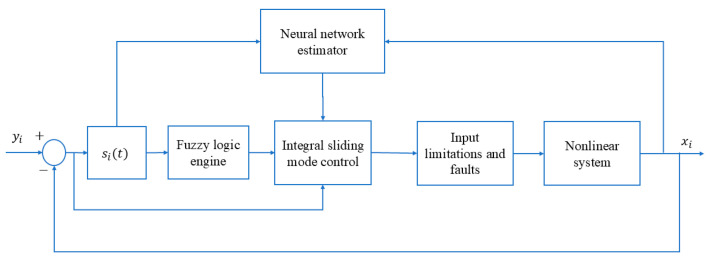
Scheme of the proposed approach, considering the challenges of control input constraints and faults.

Figure 2 showcases the block diagram of the proposed feedforward neural networks-based approach aimed at synchronizing nonlinear systems that are subject to control input limitations and faults. The proposed methodology extends several benefits over traditional control techniques. Particularly, it displays a superior capacity for managing faults and constraints within control input. Furthermore, the fuzzy engine effectively eliminates unwanted chattering and vibrations within the system.

**Remark** **2.** *The inclusion of an integral error term in our design is essential for eliminating steady-state errors, which is crucial for achieving accurate long-term performance. While this can introduce potential issues such as peaking and overshooting before reaching a steady state, these effects are carefully managed through proper tuning of the control parameters. Additionally, the fuzzy system integrated into our control design plays a pivotal role in mitigating these issues. The fuzzy logic engine dynamically adjusts the controller’s gains, which helps reduce overshoot and peaking while maintaining robust control performance. Although simpler designs could avoid these problems, they often sacrifice robustness and accuracy in the presence of disturbances or uncertainties. Our approach, enhanced by the fuzzy system, ensures both steady-state precision and adaptability while minimizing transient effects like peaking and overshooting.*

## 4. Numerical Results

In this section, we conduct an assessment of the performance of the proposed control mechanism in scenarios characterized by system faults and inherent constraints on the control inputs. The primary objective is to ascertain the controller’s capacity to handle these constraints whilst fulfilling the predefined control objectives.

We implemented the suggested controller on two distinct systems: the autonomous and the non-autonomous chaotic systems. In both instances, identical limitations and faults are consistently imposed on all control signals:

(26) $$u=\left\{\begin{array}{c} 20\;\;\;\;\;\;\;\;\;\;\;\;\;\;\;\;\;\;\;\;\;\;\;\;\;\;\;\;\;\;\;\;\;\;\;\;\;\;\;\;\;\;\;\;\;\;\;\;\;\;\;\;\;\;\;\;\;\;\;\;\;\;\;\;\;\;\mathrm{if}\;\;\;u_{a}>20\;\;\;\;\;\;\;\;\;\;\;\;\;\;\;\;\;\;\;\;\;\;\;\\ u_{a}+a_{i}\left(t\right)\left(\left(c_{i}-1\right)u_{a}+\bar{u}\right)\;\;\;\;\;\;\;\;\;\;\;\;\;\;\;\;\;\;\;\;\;\;\;\mathrm{if}-20<u_{a}<20\;\;\;\;\;\;\;\;\;\;\\ -20\;\;\;\;\;\;\;\;\;\;\;\;\;\;\;\;\;\;\;\;\;\;\;\;\;\;\;\;\;\;\;\;\;\;\;\;\;\;\;\;\;\;\;\;\;\;\;\;\;\;\;\;\;\;\;\;\;\;\;\;\;\;\;\;\;\;\mathrm{if}\;\;\;u_{a}<-20\;\;\;\;\;\;\;\;\;\;\;\;\;\;\;\;\;\;\;\;\;\;\; \end{array}\right.\;a_{i}(t)=\left\{\begin{array}{c} 0,t<t_{0}\\ 1-e_{i}^{-b_{i}(t-t_{0})}t\geq t_{0} \end{array}\right.\;t_{0}=5;c_{i}=0.8,\bar{u}=5;b_{i}=10$$

### 4.1. Example 1: Autonomous Chaotic System

In the initial example, we delve into, we focus on an autonomous chaotic system. As elucidated in [46], the chaotic dynamics of such systems were rigorously examined. Here, we introduce faults and control limitations into the system and apply our proposed methods. Equation (24) presents the state space representation of this autonomous chaotic system.

(27) $${\dot{y}}_{1}=y_{2}y_{3}\;{\dot{y}}_{2}=y_{2}^{2}-0.85y_{3}+y_{2}y_{3}\;{\dot{y}}_{3}=0.5y_{2}^{2}+my_{1}$$

The parameter m influences the behavior of the system, transitioning it from predictable to chaotic behavior. In our current study, we set m to 1, which leads to chaotic behavior. The subsequent equation represents the slave system. This formulation also incorporates considerations related to the control input:

(28) $${\dot{x}}_{1}=x_{2}x_{3}+u_{1}\;{\dot{x}}_{2}=x_{2}^{2}-0.85x_{3}+x_{2}x_{3}+u_{2}\;{\dot{x}}_{3}=0.5x_{2}^{2}+mx_{1}+u_{3}$$

**Figure 3:**
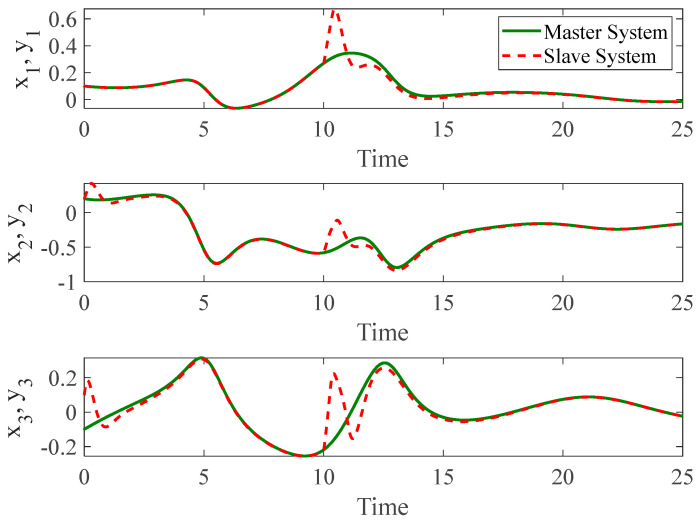
The time history of slave and master autonomous chaotic systems.

**Figure 4:**
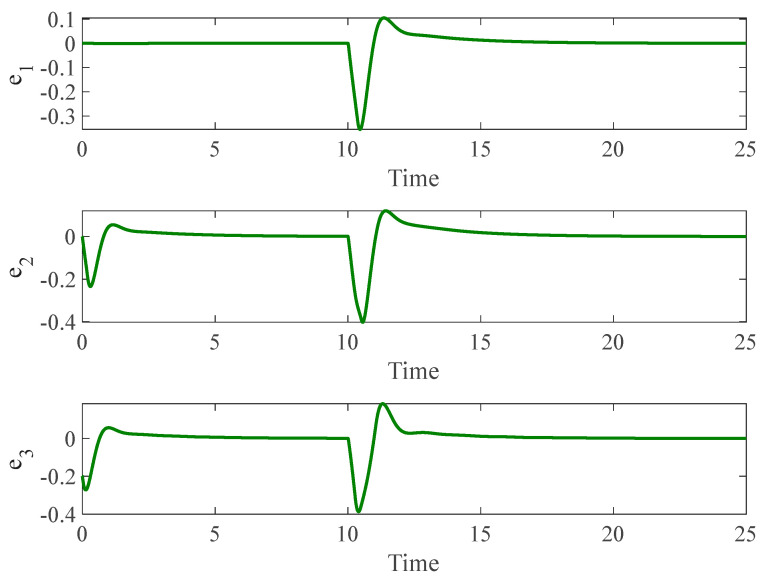
Synchronization error of autonomous chaotic systems.

**Figure 5:**
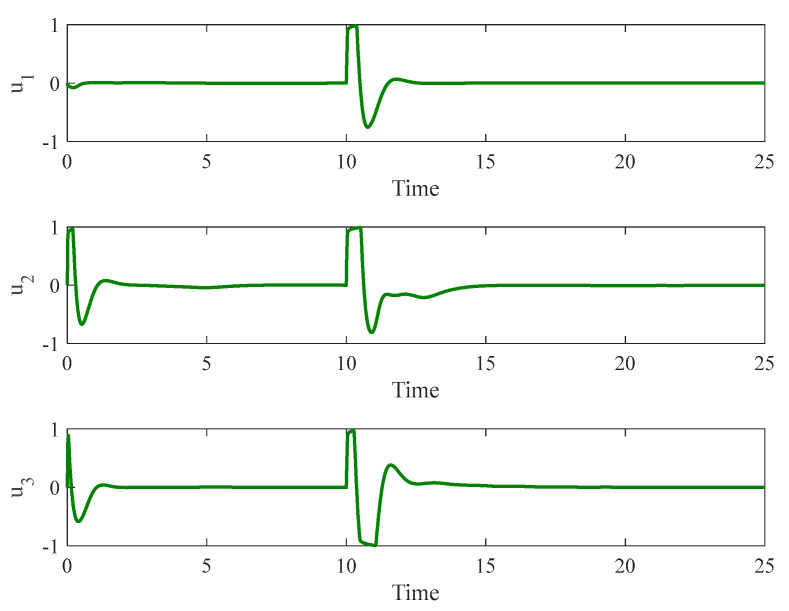
Control input for autonomous chaotic systems.

Figure 3 and Figure 4 depict the temporal progression and the associated synchronization discrepancies between the slave and master autonomous chaotic systems. Despite the introduction of a fault at t=10—evident in the aforementioned figures—the synchronization between the slave and master systems is effectively realized in a finite duration, according to the design of the proposed controller. Furthermore, Figure 5 displays the control inputs, which, due to the implemented fuzzy scheme, are noted to have appropriate magnitudes and exhibit a smooth response.

### 4.2. Example 2: None-Autonomous Chaotic System

To ensure a comprehensive examination of our control methodology, we extend its application to another system. Specifically, we apply it to a non-autonomous chaotic system. Reference [47] has delved into the intricacies of this system. Here, amidst challenging conditions marked by control input constraints and faults in the control input (described in Equation (23)), we implement our proposed strategy. Equation (26) represents the mathematical formulation of the non-autonomous chaotic system.

(29) $${\dot{y}}_{1}=y_{2}\;{\dot{y}}_{2}=y_{3}+y_{2}cos(y_{1})\;{\dot{y}}_{3}=-by_{2}+A\omega cos(\omega t)$$

The time-dependent term Aωcos(ωt) is characterized by an amplitude A and frequency ω. Equation (26) delineates the dynamics of the master system. The dynamics of the slave system, inclusive of the control input, are described in the subsequent equation:

(30) $${\dot{x}}_{1}=x_{2}+u_{1}\;{\dot{x}}_{2}=x_{3}+x_{2}cos(x_{1})+u_{2}\;{\dot{x}}_{3}=-bx_{2}+A\omega cos(\omega t)+u_{3}$$

Upon setting the forcing and inherent system parameters to A = 0.8, ω = 0.1, b = 0.7, we discern distinct types of attractors for system (26), contingent on the variation in initial conditions.

**Figure 6:**
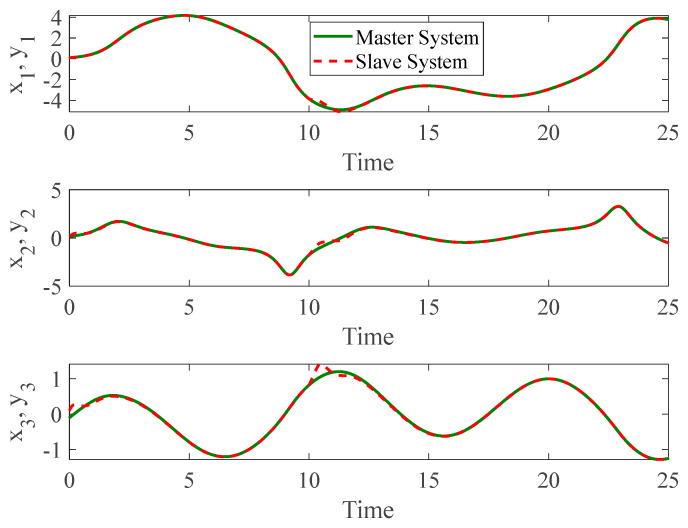
The time history of slave and master non-autonomous chaotic systems.

**Figure 7:**
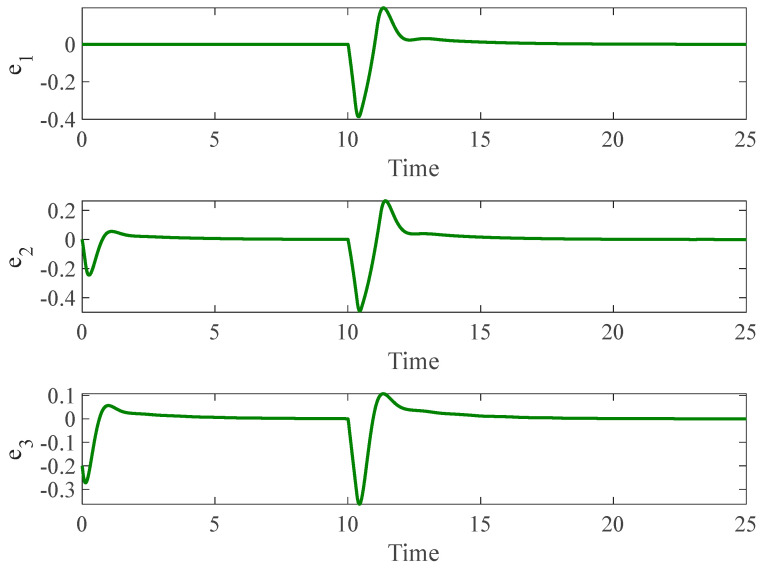
Synchronization error of non-autonomous chaotic systems.

**Figure 8:**
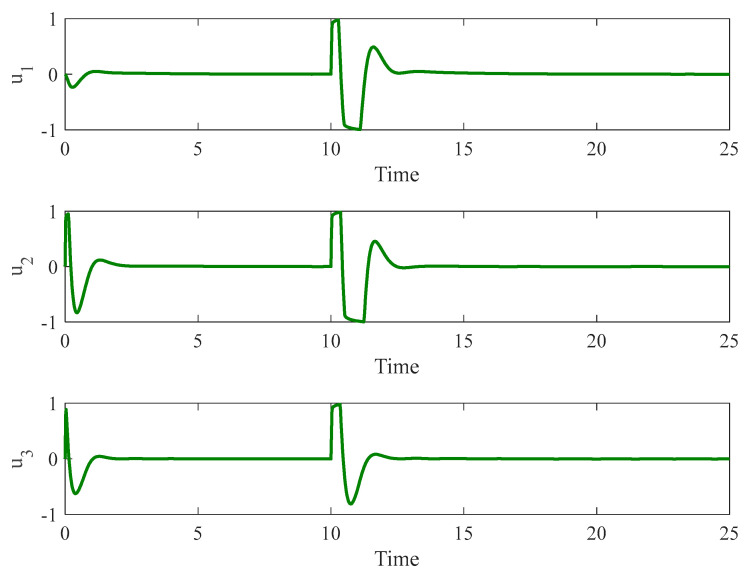
Control input for non-autonomous chaotic systems.

Figure 6 presents the temporal progression of both the slave and master non-autonomous chaotic systems, while Figure 7 elucidates the synchronization error inherent to these non-autonomous chaotic systems. Furthermore, Figure 8 illustrates the control input applied to the slave system. Notably, even when faults and control limitations pose considerable challenges, as evident in the figures at t=10, the system effectively harnesses information derived from neural networks and handles these unknown factors. This capability empowers it to adeptly manage chaotic systems even under unforeseen and demanding conditions. Moreover, our tailored controller incorporates a fuzzy decision-making system as an alternative to the sign function. It also capitalizes on the integral sliding surface to mitigate chattering—a prevalent complication encountered with traditional controllers.

### 4.3. Comparison

In this section, we compare the performance of our proposed method with a terminal sliding mode controller based on the approach presented in [48]. The terminal sliding mode controller is capable of handling non-symmetric control input saturation. To implement this controller, we fine-tuned its parameters through trial and error to achieve the lowest possible trajectory error. We performed the comparison in two stages: (1) Control Input Saturation Only and (2) Control Input Saturation with Faults which are elaborated in the following.

#### 4.3.1. Control Input Saturation Only

**Figure 9:**
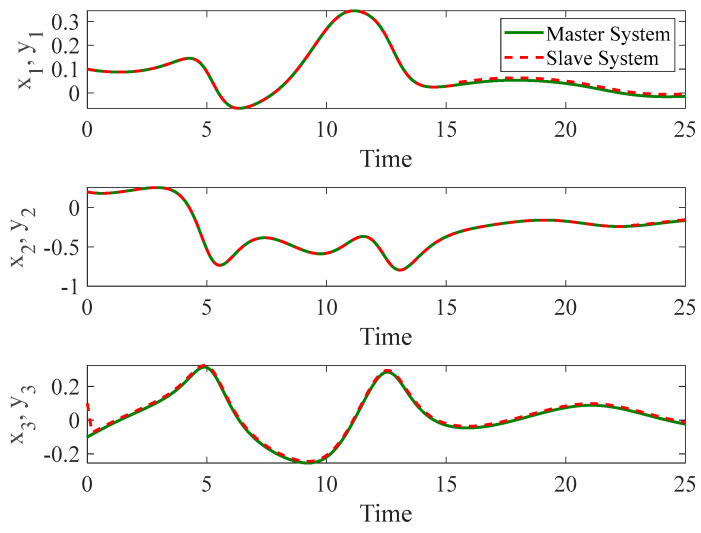
The time history of slave and master autonomous chaotic systems with control input constraints and without faults using terminal sliding mode proposed in [48].

**Figure 10:**
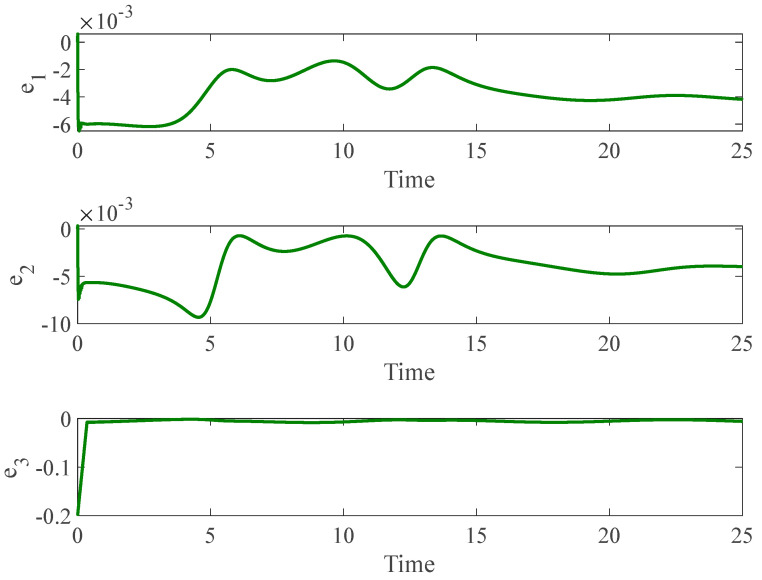
Synchronization error of autonomous chaotic systems with control input constraints and without faults using terminal sliding mode proposed in [48].

**Figure 11:**
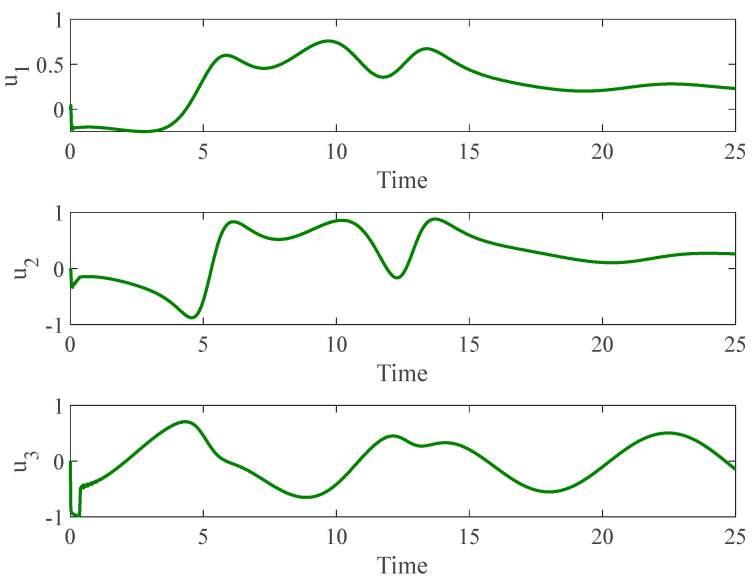
Control input for autonomous chaotic systems with control input constraints and without faults using terminal sliding mode proposed in [48].

Initially, we considered a scenario with control input saturation limits set to [−1,1]. The results, depicted in Figure 9, Figure 10 and Figure 11, demonstrate that the terminal sliding mode controller can effectively manage the input saturation and maintain system stability under these conditions.

#### 4.3.2. Control Input Saturation with Faults

**Figure 12:**
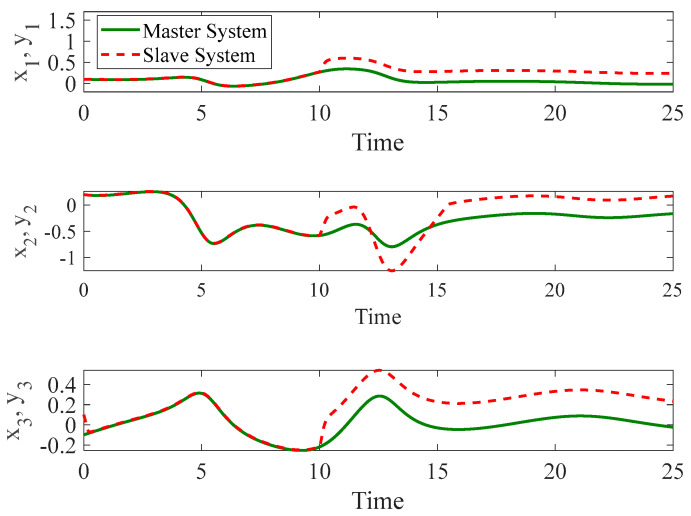
The time history of slave and master autonomous chaotic systems with both control input constraints and faults using terminal sliding mode proposed in [48].

**Figure 13:**
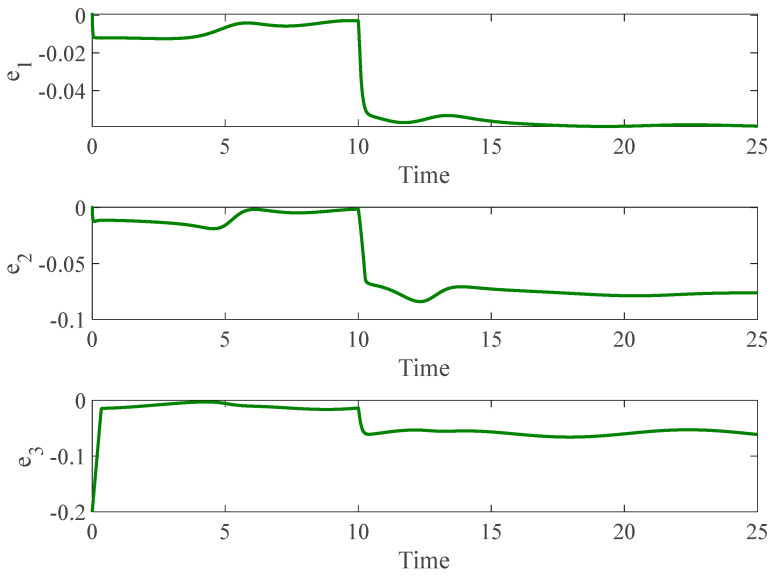
Synchronization error of autonomous chaotic systems with both control input constraints and faults using terminal sliding mode proposed in [48].

**Figure 14:**
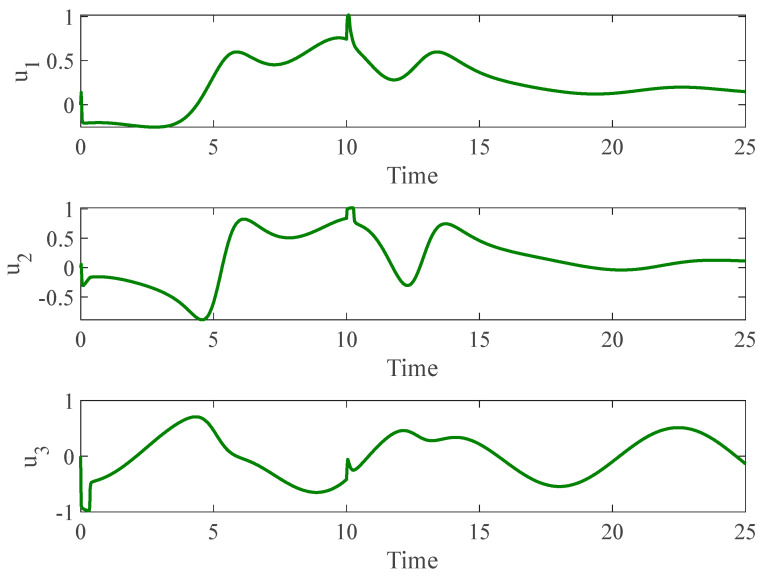
Control input for autonomous chaotic systems with both control input constraints and faults using terminal sliding mode proposed in [48].

In the second stage, we introduced the same faults as those investigated in Example 2 using a autonomous chaotic system. Figure 12, Figure 13 and Figure 14 illustrate the results under this more challenging scenario. While the terminal sliding mode controller handles bounded uncertainties well, it struggles to compensate for faults in the control input. Moreover, the presence of the sign function in the terminal sliding mode control results in chattering, which negatively affects system performance. This chattering is not observed in our proposed method due to the incorporation of a fuzzy logic system that smoothens the control response.

Overall, the comparison shows that although the terminal sliding mode controller is robust against control input saturation and bounded uncertainties, it lacks the capability to handle faults and introduces chattering due to its reliance on the sign function. Our proposed Neural Network-based Fuzzy Integral Sliding Mode Control overcomes these limitations, providing a smooth and stable response even in the presence of faults.

In summary, the findings underscore the efficacy of our proposed controller, endowed with a neural network estimator and a fuzzy logic mechanism, in synchronizing the chaotic system. The proposed combination of feedforward neural networks and fuzzy logic presents a practical approach to tackle the persistent issues in the control of nonlinear systems, paving the way for reliable and efficient control strategies.

## 5. Conclusions

In this research, we introduced a novel methodology to control and synchronize nonlinear systems, utilizing a finite-time fuzzy integral sliding mode control strategy coupled with an advanced observer. This controller is specifically designed to navigate through control input constraints and system faults while maintaining overall system stability. The proven finite-time convergence of the sliding surface ensures rapid stabilization of the system states. A distinctive feature of this controller is its integration with a neural network-based observer, which detects and compensates for faults and limitations in the control actuator. This is further enhanced by the incorporation of a fuzzy logic mechanism, which smooths the control response and minimizes oscillations. Additionally, we compared the proposed method with a terminal sliding mode controller and demonstrated that our approach effectively handles system faults, which the terminal sliding mode control cannot manage. The proposed method was evaluated on both non-autonomous and autonomous chaotic systems under challenging conditions. The simulation results validate the controller’s ability to synchronize the systems and achieve the desired performance, even in the presence of control input constraints and faults. These findings highlight the potential of the proposed methodology as a robust solution for controlling highly nonlinear systems in complex environments. Future work could focus on refining the convergence of the sliding surface $s_{i}(t)$ itself using finite-time techniques, as opposed to its current exponential behavior. This enhancement would potentially improve the speed and robustness of the overall control system.

## Figures and Tables

**Table 1:** Fuzzy rule base for $F_{s}$.

	$\dot{s}$					
s	$F_{s}$	NB	N	Z	P	PB
	NB	NB	NB	N	NB	NB
	N	NB	N	N	N	NB
	Z	N	N	Z	P	P
	P	PB	P	P	P	PB
	PB	PB	PB	P	PB	PB

## Data Availability

Dataset available on request from the authors.
